# Refractory adult Coats disease treated with dexamethasone intravitreal implant: A case report

**DOI:** 10.1097/MD.0000000000020249

**Published:** 2020-05-15

**Authors:** Yu-hua Ding, Bang-tao Yao, Xiao-gui Zhao, Hao Yu, Gang Liu, Xiu-ying Wang

**Affiliations:** a Department of Ophthalmology, Jiangsu Province Hospital, The First Affiliated Hospital of Nanjing Medical University; b Department of Ophthalmology, Lishui District People's Hospital, Lishui branch of Southeast University Affiliated Zhongda Hospital, Nanjing, Jiangsu Province, China.

**Keywords:** Coats disease, dexamethasone intravitreal implant, exudative retinal detachment, macular edema, visual acuity

## Abstract

**Introduction:**

Coats disease is a sporadic, retinal vascular abnormality, causing blindness. Several interventional methods, including laser photocoagulation, have been proposed; however, the use of intravitreal dexamethasone in refractory Coats disease is not well described.

**Patient concerns:**

A 38-year-old man presented with a painless reduction in visual acuity in his right eye, commencing 15 days prior to initial assessment.

**Diagnosis:**

Clinical manifestations and multimodal imaging indicated Coats disease.

**Interventions:**

Retinal laser photocoagulation was performed in the nonperfused areas, 15 months later, the exudative retinal detachment, and macular edema remained, the patient was then treated with an intravitreal slow-release dexamethasone implant.

**Outcomes:**

The exudative retinal detachment and macular edema had resolved, and the BCVA had also improved.

**Conclusion:**

Dexamethasone intravitreal implantation was effective in treating refractory Coats disease.

## 1 Introduction

Coats disease, first described by George Coats in 1908, is a sporadic, retinal vascular abnormality, characterized by telangiectasia and exudate accumulation.^[1]^ It is mostly unilateral and occurs in children, however, the disease etiology remains unclear. Several complications of Coats disease, such as exudative retinal detachment, macular edema, and neovascular glaucoma, have been reported.^[2]^ Previously described treatments, include retinal laser photocoagulation, intravitreal injection of triamcinolone acetonide or anti-vascular endothelial growth factor (VEGF); however, several consequences of these modalities have also been reported.^[3,4,5]^ Here we reported a refractory case of adult-onset Coats disease, treated with an intravitreal implant, releasing dexamethasone, following ineffective laser photocoagulation therapy.

## 2 Case presentation

**Figure 1:**
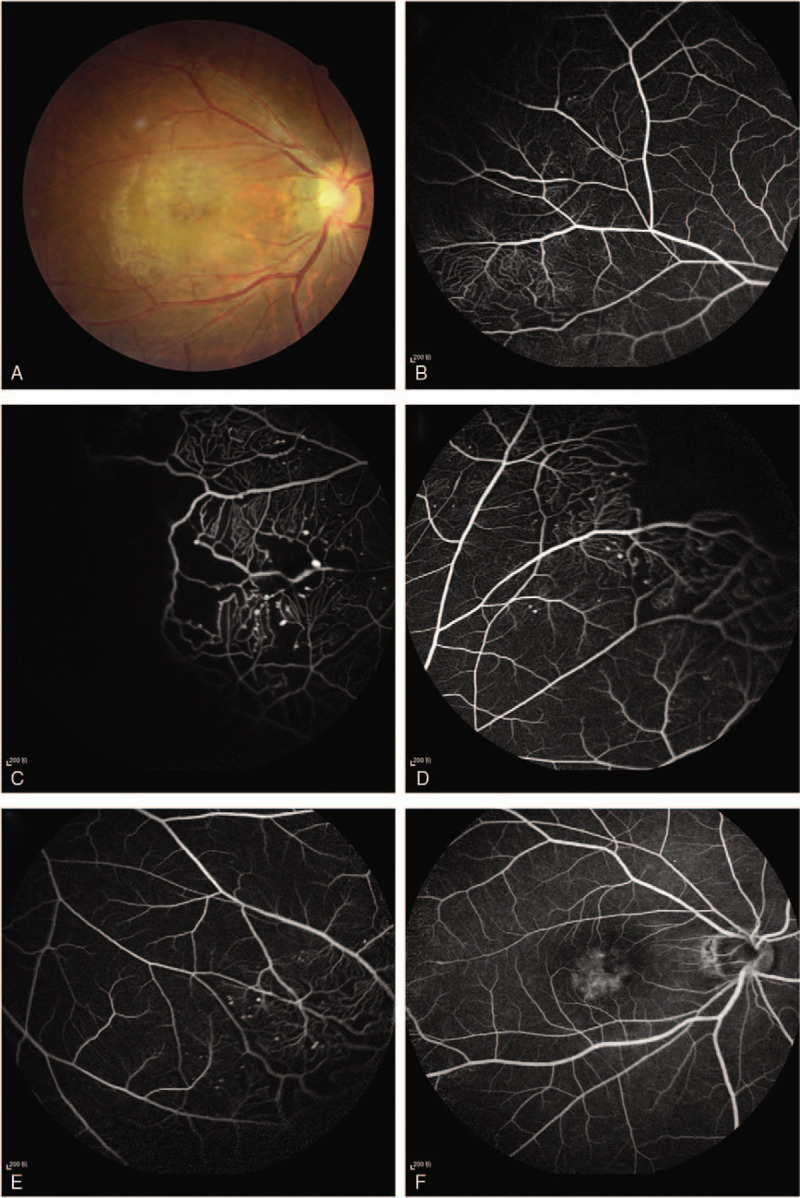
(A) Fundus examination revealed scattered exudate in the macula of the right eye; (B-F) FFA revealed an area of telangiectatasia, aneurysms and retinal nonperfusion in the peripheral quadrants, with foveal hyper-fluorescence in the late phase.

**Figure 2:**
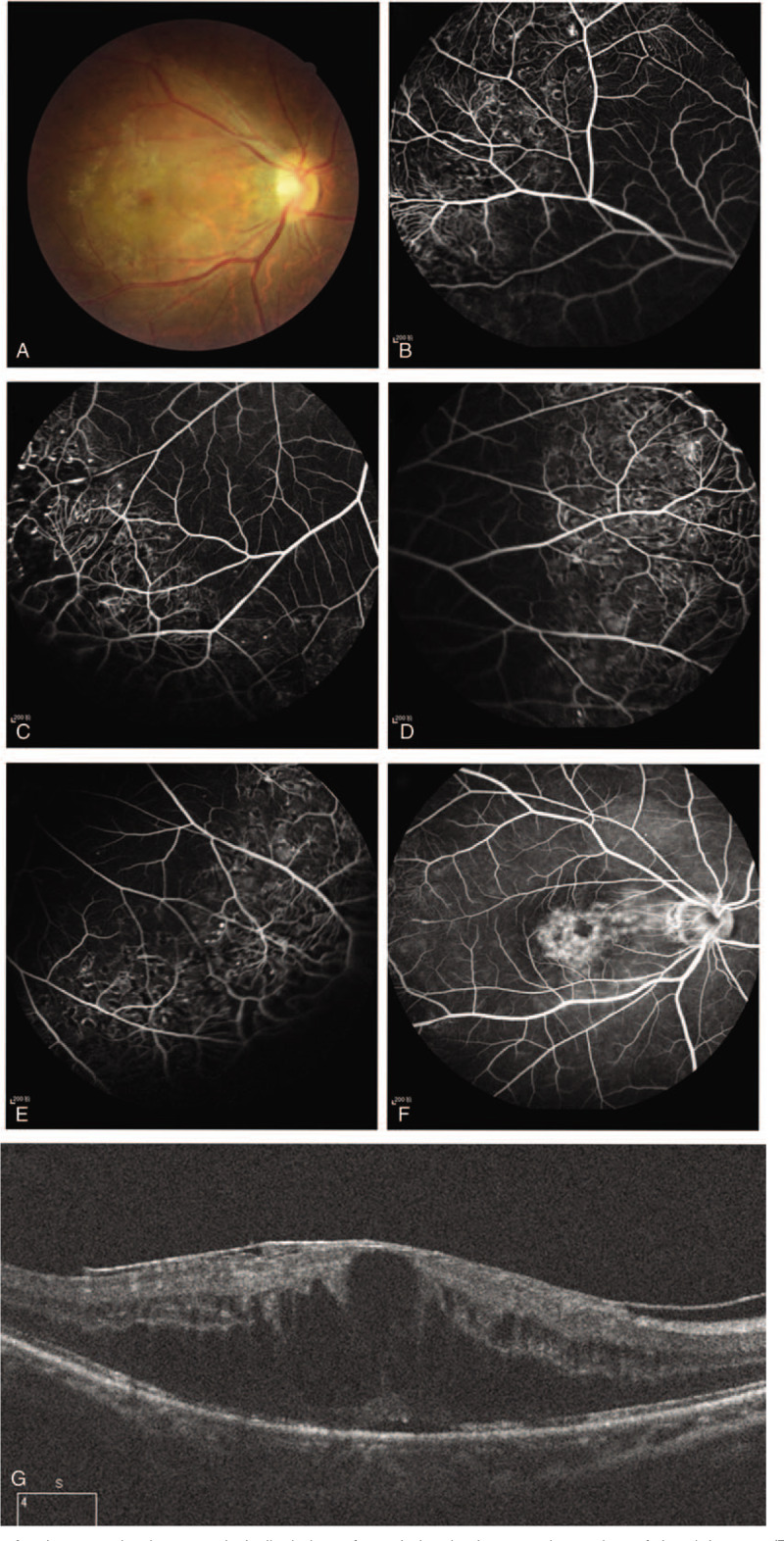
(A) 3-month follow-up fundus examination revealed alleviation of exudation in the macular region of the right eye; (B-F) FFA demonstrated retinal telangiectatic vessels, with focal slightly increased hyper-fluorescence at the fovea in the late phase; (G) SD-OCT displayed the presence of exudative retinal detachment and macular edema.

A 38-year-old man presented with a painless reduction in visual acuity in his right eye, commencing 15 days prior to initial assessment. His medical and ophthalmic history were unremarkable. The best-corrected visual acuity (BCVA) in the right eye was 20/30, and 20/20 in the left eye, the anterior segments were clear, and the intraocular pressure was normal in both eyes. Fundus examination revealed scattered exudate in the macula of the right eye (Fig. 1A). Fundus fluorescein angiography (FFA) revealed an area of telangiectatasia, aneurysms and retinal nonperfusion in the peripheral quadrants, with foveal hyper-fluorescence in the late phase (Fig. 1B-F), leading to a diagnosis of Coats disease. Retinal laser photocoagulation was performed in the nonperfused areas over 4 sessions, at 7-day intervals. Minimal improvement was seen after 3 months (Fig. 2), and further laser photocoagulation given.

**Figure 3:**
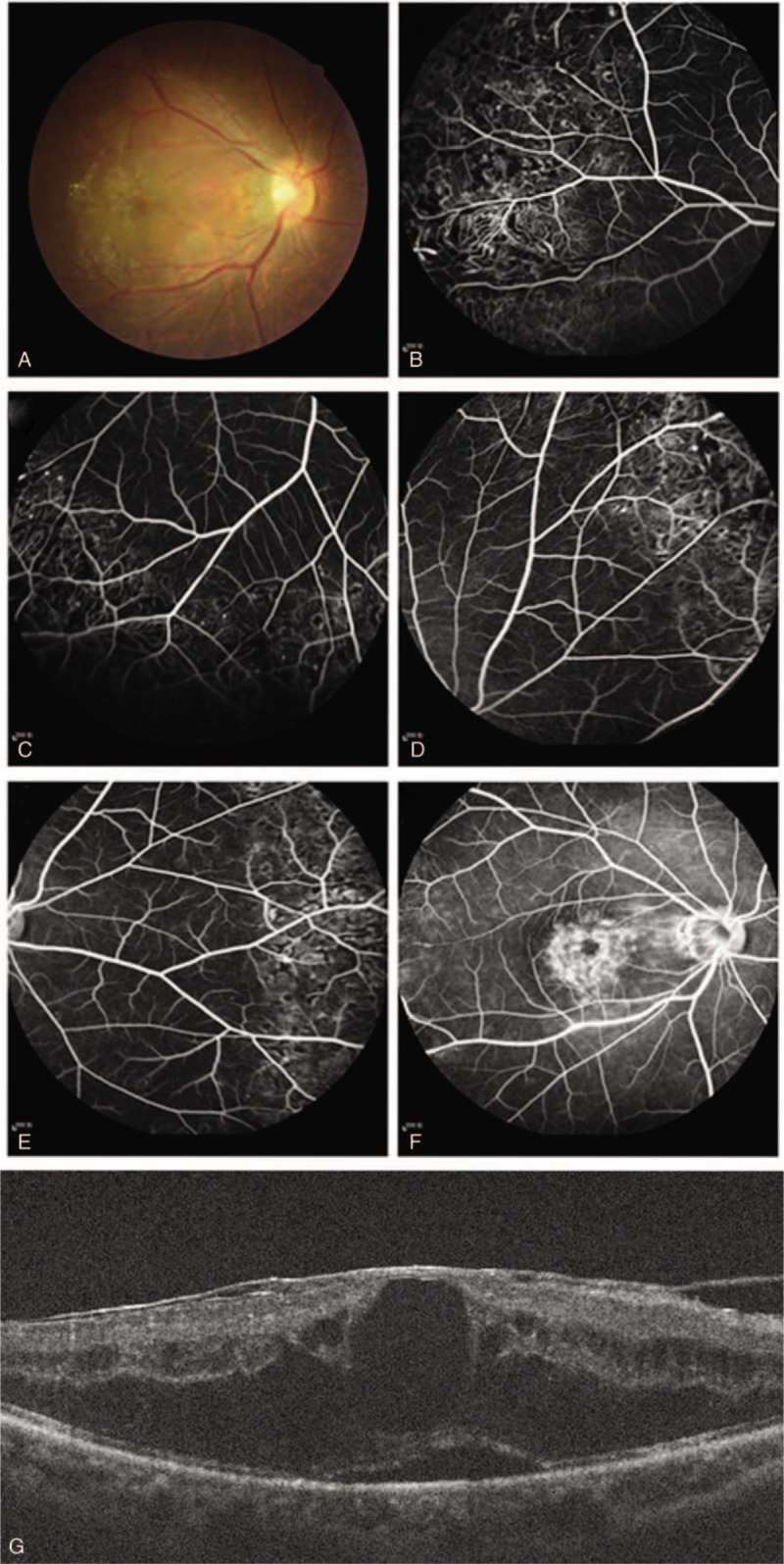
(A) 15-month follow-up fundus examination revealed exudation in the temporal macula of the right eye; (B-F) FFA revealed scarring and foveal fluorescence accumulation during the late phase; (G) SD-OCT revealed that the exudative retinal detachment and macular edema seen at 3 months, remained.

After 15 months, the BCVA was reduced to 20/125. Fundus examination revealed exudation in the temporal macula of the right eye (Fig. 3A), and FA revealed scarring and foveal fluorescence accumulation during the late phase (Fig. 3B-F). Spectral-domain optical coherence tomography (SD-OCT) revealed that the exudative retinal detachment and macular edema seen at 3 months, remained (Fig. 3G).

**Figure 4:**
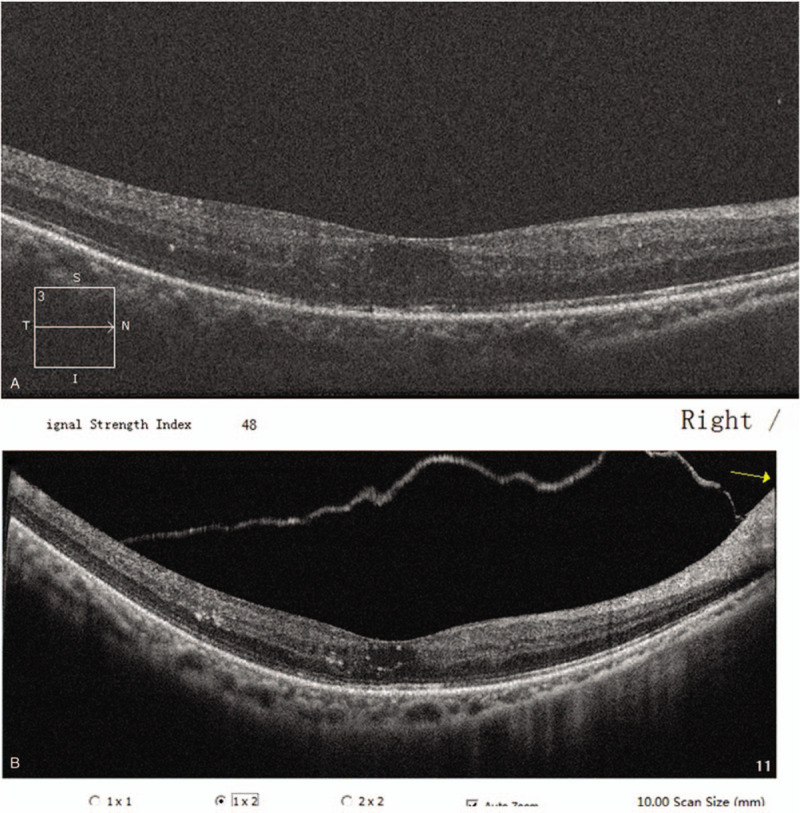
(A) After 2 months, the exudative retinal detachment and macular edema had resolved; (B) These improvements persisted for at least 4 months.

The patient was then treated with an intravitreal slow-release dexamethasone implant (Ozurdex, Allergan, Inc., Irvine, CA, USA). After 2 months, the exudative retinal detachment and macular edema had resolved (Fig. 4A), and the BCVA had also improved to 20/30. These improvements persisted for at least 4 months (Fig. 4B).

## 3 Discussion and conclusions

Coats disease is a retinal disorder characterized by retinal telangiectasia, leading to exudate accumulation, exudative retinal detachment, macular edema, and/or secondary glaucoma.^[2]^ The etiology of the disease is unclear, and the prognosis remains poor. Currently, no guidelines exist for treating Coats disease, although, several interventional methods have been proposed, including laser photocoagulation, anti-VEGF therapy, surgery, and intravitreal injection of triamcinolone acetonide.^[5,6,7,8]^

Retinal laser photocoagulation can destroy the affected retinal blood vessels, particularly the telangiectasias and aneurysms.^[6]^ However, exudative retinal detachment may remain unresolved, due to the presence of subretinal fluid.^[2]^ Therefore, close observation is required in order to detect the possible recurrence of subretinal detachment. In this case, retinal laser photocoagulation was performed to treat the nonperfused areas. Declining exudation and peripheral retinal scars were observed at the 15-month follow-up, but the exudative retinal detachment and macular edema remained, leading us to consider it a refractory case.

Alleviation of macular edema and exudation, with complete resolution of exudative retinal detachment, has been shown with intravitreal anti-VEGF therapy.^[7]^ The immediate shrinkage of the proliferative membrane, and the improvement of secondary tractional retinal detachment has also been seen, following bevacizumab injection.^[4]^ Intravitreal triamcinolone acetonide can also be used to promote absorption of the subretinal fluid and exudate, however, this has also been associated with elevated intraocular pressure, cataract, and endophthalmitis.^[5]^

In clinically advanced cases of Coats disease, surgical interventions may alleviate the significant epiretinal traction caused by the preretinal membranes or proliferative vitreoretinopathy. Vitrectomy can eliminate the VEGF-containing vitreous; however, it may result in iatrogenic retinal breaks.^[8]^ The resolution of fibrosis and traction favors the treatment of telangiectasia and exudative retinal detachment by retinal laser photocoagulation and/or cryotherapy.

Ozurdex is a biodegradable, sustained-release dexamethasone delivery system, used to treat macular edema secondary to diabetic retinopathy or age-related macular degeneration; however, few cases describing the therapeutic effects of Ozurdex in refractory Coats disease have been reported. Saatci et al demonstrated that Ozurdex implantation contributed to favorable anatomical and visual outcomes in patients with Coats disease.^[9]^ The effects of anti-VEGF drugs only last 1 month; hence, monthly intravitreal injections are required, potentially increasing patient discomfort and the risk of infection. The effects of Ozurdex persist for up to 6 months, meaning Ozurdex is a safe and more convenient treatment option than the anti-VEGF drugs.^[9]^ In this study, treatment with Ozurdex resolved retinal detachment and macular edema, improved visual acuity to 20/30, and normalized intraocular pressure, with no further recurrence noted during the 4-month follow-up period.

In conclusion, Coats disease is a sporadic, retinal vascular abnormality, associated with several serious complications. The use of an intravitreal dexamethasone implant has been shown to be an effective method for treating the complications in refractory Coats patients. Further studies involving more cases and longer follow-up periods are required in order to better understand the efficacy and safety of therapy with Ozurdex.

## Author contributions

**Investigation:** Bangtao Yao

**Project administration:** Yuhua Ding

**Supervision:** Xiaogui Zhao, Hao Yu

**Writing – original draft:** Bangtao Yao, Xiaogui Zhao, Yuhua Ding

**Writing – review & editing:** Gang Liu, Xiuying Wang

All authors read and approved the final manuscript.
